# *Staphylococcus aureus* SrrAB Affects Susceptibility to Hydrogen Peroxide and Co-Existence with *Streptococcus sanguinis*

**DOI:** 10.1371/journal.pone.0159768

**Published:** 2016-07-21

**Authors:** Yuichi Oogai, Miki Kawada-Matsuo, Hitoshi Komatsuzawa

**Affiliations:** Department of Oral Microbiology, Kagoshima University Graduate School of Medical and Dental Sciences, Kagoshima, Japan; Rockefeller University, UNITED STATES

## Abstract

*Staphylococcus aureus* is a pathogen and a commensal bacterial species that is found in humans. Bacterial two-component systems (TCSs) sense and respond to environmental stresses, which include antimicrobial agents produced by other bacteria. In this study, we analyzed the relation between the TCS SrrAB and susceptibility to the hydrogen peroxide (H_2_O_2_) that is produced by *Streptococcus sanguinis*, which is a commensal oral streptococcus. An *srrA*-inactivated *S*. *aureus* mutant demonstrated low susceptibility to the H_2_O_2_ produced by *S*. *sanguinis*. We investigated the expression of anti-oxidant factors in the mutant. The expression of *katA* in the mutant was significantly higher than in the wild-type (WT) in the presence or absence of 0.4 mM H_2_O_2_. The expression of *dps* in the mutant was significantly increased compared with the WT in the presence of H_2_O_2_ but not in the absence of H_2_O_2_. A *katA* or a *dps*-inactivated mutant had high susceptibility to H_2_O_2_ compared with WT. In addition, we found that the nitric oxide detoxification protein (flavohemoglobin: Hmp), which is regulated by SrrAB, was related to H_2_O_2_ susceptibility. The *hmp*-inactivated mutant had slightly lower susceptibility to the H_2_O_2_ produced by *S*. *sanguinis* than did WT. When a *srrA*-inactivated mutant or the WT were co-cultured with *S*. *sanguinis*, the population percentage of the mutant was significantly higher than the WT. In conclusion, SrrAB regulates *katA*, *dps* and *hmp* expression and affects H_2_O_2_ susceptibility. Our findings suggest that SrrAB is related *in vivo* to the co-existence of *S*. *aureus* with *S*. *sanguinis*.

## Introduction

*Staphylococcus aureus* is a human pathogen that causes several diseases such as suppurative diseases, food poisoning and toxic shock syndrome [1, 2]. Recently, methicillin-resistant *S*. *aureus* epidemics in hospitals have become a worldwide health problem [3–5]. *S*. *aureus* is a commensal bacterium found in humans that has been isolated from the skin and nasal mucosa of healthy subjects with a frequency of 20 to 60% [6, 7]. Additionally, *S*. *aureus* is known to inhabit the oral cavity, including the oral mucosa, gingiva and dental plaque [8–10].

In a commensal bacterial flora, many bacteria produce anti-bacterial agents such as bacteriocins [11, 12] and hydrogen peroxide compete with other bacterium [13–15]. It was demonstrated in virginal flora that H_2_O_2_-producing lactobacilli inhibited the growth of pathogens [16, 17]. In oral flora, viridans group streptococci produced H_2_O_2_ and had an antagonistic effect on pathogens [13–15]. *Streptococcus sanguinis* is an oral bacterium that is found primarily in dental plaques and has been reported to be an H_2_O_2_-producing species. Several reports have demonstrated that the H_2_O_2_ produced by *S*. *sanguinis* can kill other oral bacterial species [18, 19]. Uehara *et al*. reported that viridans group streptococci containing *S*. *sanguinis* inhibit colonization with *S*. *aureus* in newborns, which has been attributed to H_2_O_2_ [20, 21]. On the other hand, *S*. *aureus* was reported to possess several factors that confer resistance to H_2_O_2_, such as catalase (KatA), alkyl hydroperoxide reductase (AhpC) and DNA-binding proteins from starved cells (Dps) [22–24]. KatA and AhpC are enzymes that decompose H_2_O_2_. Dps is an inhibitor of hydroxyl radical (·OH) production from H_2_O_2_ in the presence of iron via the Fenton chemistry. Therefore, the biological relevance of interactions between *S*. *sanguinis*, a resident of the oral cavity, and *S*. *aureus* is uncertain.

Two-component systems (TCSs) are composed of a sensor kinase and a response regulator and are bacterial-specific gene regulation systems. When a sensor kinase senses a stimulant in the extracellular environment, the response regulator is phosphorylated and regulates several genes to facilitate adaptation to the environment [25]. Recently, several TCSs have been reported to be important for adaptation to H_2_O_2_ stress. In *Escherichia coli*, *Salmonella enterica* Serovar Typhimurium and *Haemophilus influenzae*, ArcAB has an oxygen sensing function and is essential for resisting reactive oxygen species, including H_2_O_2_ [26–28]. In *S*. *aureus*, Sun *et al*. demonstrated that two TCSs (AgrCA and AirSR) affected the susceptibility to H_2_O_2_ [29, 30].

The TCS SrrAB is a known oxygen sensor in *S*. *aureus* and regulates several virulence genes under low oxygen conditions [31–33], as well as anaerobic metabolism genes and a flavohemoglobin *hmp* under low oxygen conditions or upon exposure to nitric oxide (NO) [34, 35]. However, the relation between susceptibility to H_2_O_2_ and SrrAB is unknown. In this study, we investigated the effects of SrrAB on susceptibility to the H_2_O_2_ produced by *S*. *sanguinis*.

## Materials and Methods

### Bacterial strains and growth conditions

The bacterial strains used in this study are listed in Table 1. *S*. *aureus* was grown in 5 ml of tryptic soy broth (TSB) (Becton Dickinson Microbiology Systems, Cockeysville, MD, USA) in test tubes (18 mm diameter × 150 mm tall) at 37°C under aerobic conditions with shaking (120 rpm). *S*. *sanguinis* was aerobically grown in 5 ml of TSB in test tubes (18 mm diameter × 150 mm tall) at 37°C under 5% CO_2_ without shaking. Tetracycline (Tc; 5 μg / ml) and chloramphenicol (Cp; 3 μg / ml) were added for the maintenance of *S*. *aureus* mutant strains. Ampicillin (100 μg / ml) and spectinomycin (50 μg / ml) were added for the maintenance of *E*. *coli* mutant strains.

**Table 1:** Strains and plasmids used in this study.

Strains or plasmids	Description	References or source
*Staphylococcus aureus*		
MW2	Clinical strain, sepsis, methicillin resistant (*mecA*+)	[59]
TY34	Clinical strain, impetigo, methicillin resistant (*mecA*+)	[37]
RN4220	Restriction-deficient transformation recipient	[40]
MW2 Δ*srrA*	*srrA*::pCL52.1 in MW2, Tc^r^	[36]
MW2 *srrAB* compl.	*srrAB* complemented in MW2 Δ*srrA* by pYO10, Tc^r^ Cp^r^	This study
TY34 Δ*srrA*	*srrA*::pCL52.1 in TY34, Tc^r^	[37]
TY34 *srrAB* compl.	*srrAB* complemented in TY34 Δ*srrA* by pYO10, Tc^r^ Cp^r^	This study
MW2 Δ*hmp*	*hmp*::pCL52.1 in MW2, Tc^r^	This study
MW2 Δ*dps*	*dps*::pCL52.1 in MW2, Tc^r^	This study
MW2 Δ*katA*	*katA*::pCL52.1 in MW2, Tc^r^	This study
MW2 Δ*perR*	*perR*::pCL52.1 in MW2, Tc^r^	This study
MW2::pCL8	MW2 harbouring pCL8, Cp^r^	This study
*Streptococcus sanguinis*		
GTC217	Ofloxacin resistance	GTC
*Escherichia coli*		
XLII-Blue	*endA1 supE44 thi-1 hsdR17 recA1 gyrA96 relA1 lac* [F’ *proAB lacI*q*ZΔM15* Tn*10* (Tet^r^) Amy Cam^r^]	Stratagene
Plasmids		
pCL52.1	*E*. *coli*–*S*. *aureus* shuttle vector, thermosensitive replicon of pE194, Tc^r^ (*S*. *aureus*), Spc^r^ (*E*. *coli*)	[39]
pCL8	*E*. *coli*–*S*. *aureus* shuttle vector, Cp^r^ (*S*. *aureus*), Amp^r^ (*E*. *coli*)	[39]
pYO10	pCL8 containing a PCR fragment of *srrAB* for complementation	This study

Tc^r^, resistant to tetracycline; Cp^r^, resistant to chloramphenicol; Spc^r^, resistant to spectinomycin; Amp^r^, resistant to ampicillin; GTC, gifu type culture.

### Construction of *S*. *aureus* mutants

The *srrA*-inactivated mutants were previously constructed [36, 37]. The genes *dps*, *katA*, *hmp* and *perR* were inactivated in *S*. *aureus* strain MW2 using the thermosensitive plasmid pCL52.1 by a previously described method [38]. Gene complementation was performed in the *srrA*-inactivated mutants using pCL8, which is an *E*. *coli*-*S*. *aureus* shuttle vector [39]. Entire sequences of *srrAB* with their own promoters were amplified by PCR. The amplified DNA was cloned into the pCL8 vector using *E*. *coli* XLII-Blue cells. The constructs were purified and electroporated into *S*. *aureus* RN4220, which was the recipient for the foreign plasmid [40]. Then, the plasmid was transduced into the mutant strains using the phage 80 alpha [41]. As a control strain for co-culture assays, strain MW2 harbouring the empty pCL8 was constructed. The primers used are listed in Table 2.

**Table 2:** Primers used in this study.

Gene name	Forward primer	Reverse primer
For gene inactivation		
*hmp*	5’- TTCAAGCTTGGGCAAAAGCATATGGCG	5’- GCGGGATCCTGATGGCTTGCGATACTG
*dps*	5’- GTTAAGCTTGAATTGAATCAACAAGTAGC	5’- TTAGGATCCTCTACTGATGTTTGCATACC
*katA*	5’- AAAAAGCTTCTGAAATAGGTAAGCAAACC	5’- AATGGATCCTCTTTATGGTTTTTAGCTTG
*perR*	5’- ACAAAGCTTAGACAAGCAATATTACG	5’- AAAGGATCCCATATGCTGAGCTAATC
For complementation		
*srrAB*	5’- TTAGGATCCGTATGCGCTTTCCTGTG	5’- AGTGGATCCTCAATAACATGCGTTCTG
For quantitative PCR		
16s rRNA	5’- CCTTATGATTTGGGCTAC	5’- TACAATCCGAACTGAGAACA
*katA*	5’- AAAGGTTCTGGTGCATTTGG	5’- AACGCAAATCCTCGAATGTC
*ahpC*	5’- TTATCGACCCAGACGGTGTT	5’- TAGCGCCTTCTTCCCATTTA
*dps*	5’- CGGTAGGAGGAA ACCCTGTA	5’- TGATACATCATCGCCAGCAT
*hmp*	5’- AAGGCTATATTGGCGCTGAA	5’- TGCAACGCTTAGTCTTGGAA
*cidA*	5’—TAGCCGGCAGTATTGTTGGT	5’—AATTTCGGAAGCAACATCCA
*perR*	5’—ACAAGCAGGCGTAAGAAT	5’—GTCGCAACACTTATATTTGG

Restriction sites are underlined.

### Direct assay for evaluating susceptibility to H_2_O_2_ produced by *S*. *sanguinis*

The direct assay method was modified from a previously described method [42]. A total of 5 μl of *S*. *sanguinis* (10^8^ cells / ml) was dropped onto a tryptic soy agar (TSA) plate. After 16 h of aerobic incubation at 37°C under 5% CO_2_, the mid-log phase (cell density 660 nm = 0.8) of *S*. *aureus* strains (10^7^ cells) mixed with 6 ml of pre-warmed tryptic soy soft agar (0.5% agar) was poured over the plates. The plates were incubated overnight at 37°C under aerobic conditions. To analyze the effects of anaerobic conditions on the production of an antibacterial agent, *S*. *sanguinis* was grown on TSA plates anaerobically using a GasPak system (Mitsubishi Gas Chemical Company Inc., Tokyo, Japan). Then, after pouring tryptic soy soft agar containing *S*. *aureus*, the plate was incubated overnight at 37°C under anaerobic conditions. To neutralize the H_2_O_2_ produced by *S*. *sanguinis*, 20 μl of bovine liver catalase (100 μg / ml) (Sigma-Aldrich, St. Louis, MO, USA) was dropped onto the area surrounding the *S*. *sanguinis* colony, and the direct assay was performed under aerobic conditions. The diameter of the *S*. *aureus* inhibition zone was measured in three directions to evaluate the inhibitory size. Three independent experiments were performed and are expressed as the mean ± SD.

### H_2_O_2_ susceptibility test

Mid-log phase (cell density at 660 nm = 0.8) *S*. *aureus* strains were washed with PBS and re-suspended in TSB. Then, 0.5 × 10^8^ cells were inoculated into 10 ml of TSB or TSB containing 0.4 mM H_2_O_2_ in a test tube (18 mm diameter × 150 mm tall) and grown aerobically at 37°C with shaking at 120 rpm. Bacterial growth was monitored to measure the bacterial density (OD 660 nm) for 2 to 10 h using the spectraphotometer miniphoto 518R (Taitec Corporation, Saitama, Japan).

### Quantitative PCR

A small amount of the *S*. *aureus* strains (10^8^ cells) was inoculated in 10 ml of TSB and grown aerobically to mid-log phase (cell density at 660 nm = 0.8) at 37°C with shaking at 120 rpm. The cultures were transferred to a centrifuge tube and treated with or without 0.4 mM H_2_O_2_ for 10 min at 37°C with shaking (120 rpm). RNA extraction was performed using a FastRNA Pro Blue Kit (MP Biomedicals, Santa Ana, CA, USA) according to the manufacturer’s protocol. One microgram of total RNA was reverse-transcribed into cDNA using a Transcriptor First Strand cDNA Synthesis Kit (Roche Diagnostics, Basel, Switzerland). Using cDNA as the template, quantitative PCR was performed using a LightCycler Nano (Roche Diagnostics). The primers used are listed in Table 2. Transcriptional levels were determined using 2^-ΔΔCt^ methods [43]. The Ct value of 16S rRNA in 1000-fold diluted cDNA was used as a reference. The means of Ct values in WT untreated with H_2_O_2_ (*N* = 5) were used as the calibrator. All test and calibrator samples were normalized to the ΔCt value (ΔCt_(test)_ = Ct_(target test)_-_(reference test)_, ΔCt_(calibrator)_ = Ct_(target calibrator)_-_(reference calibrator)_. Then, the ΔΔCt value was determined (ΔΔCt = ΔCt_(test)_-ΔCt_(calibrator)_). The relative expression level was calculated using the formula *F* = 2^-ΔΔCt^. Individual experiments were performed three or five times, and the results expressed as the mean ± SD.

### Co-culture assay

Co-culture assays were performed using a previously described method [42]. Mid-log phase cells (cell density at 660 nm = 0.8) of the *S*. *sanguinis* and *S*. *aureus* strains were adjusted to 2 × 10^8^ cells / ml using PBS. The same volume of *S*. *aureus* and *S*. *sanguinis* was mixed and 20 μl of the mixture was dropped onto a TSA plate. The plate was incubated for 2 h at 37°C under 5% CO_2_. The agar in the spotted area was excised and incorporated into 500 μl of PBS. Then, the agar was vigorously mixed to detach the bacterial cells from the agar. Appropriate dilutions were plated on TSA plates containing Cp (3 μg / ml), Tc (5 μg / ml), or ofloxacin (Oflx) (1 μg / ml) because of different susceptibilities to antibiotics. WT *S*. *aureus* (MW2::pCL8) were selected with Cp. *S*. *aureus* mutants, and complemented strains were selected with Tc. *S*. *sanguinis* was selected with Oflx. After an overnight incubation at 37°C under 5% CO_2_, CFUs were determined, and the population percentage for each *S*. *aureus* strain was calculated. To analyze the effects of pre-culturing *S*. *sanguinis*, ten microliters of *S*. *sanguinis* (10^8^ cells / ml) was dropped onto a TSA plate and the plate was incubated for 1 h at 37°C under 5% CO_2_. Then, ten microliters of *S*. *aureus* (10^8^ cells / ml) was dropped onto a *S*. *sanguinis* colony and the plate was incubated for 2 h at 37°C under 5% CO_2_. The population percentage of *S*. *aureus* was determined by the method described above. Three independent experiments were performed and the results are expressed as the mean ± SD.

### Statistical analysis

All statistical analyses were performed with statistical software EZR version 1.32 (http://www.jichi.ac.jp/saitama-sct/SaitamaHP.files/statmedEN.html).

## Results

### Susceptibility of the *srrA*-inactivated mutants to H_2_O_2_ produced by *S*. *sanguinis*

**Fig 1:**
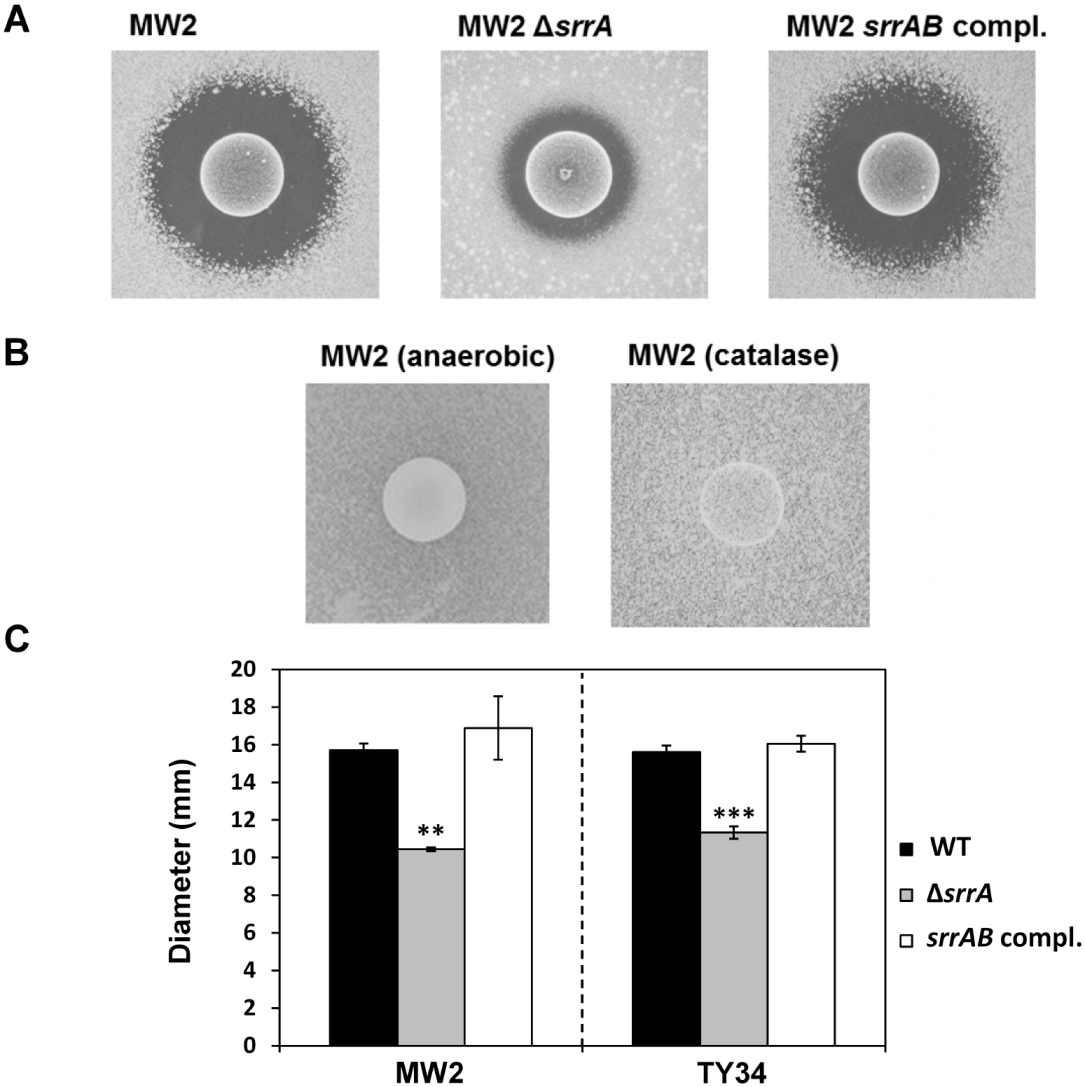
Susceptibility of the *S*. *aureus srrA*-inactivated mutant to the H_2_O_2_ produced by *S*. *sanguinis*. (A) The susceptibilities of the *S*. *aureus* MW2 WT, MW2 *srrA*-inactivated mutant and the complemented strain to the H_2_O_2_ produced by *S*. *sanguinis* were analyzed by direct assay, as described in the Materials and Methods section. (B) The susceptibility of MW2 WT to the H_2_O_2_ produced by *S*. *sanguinis* was determined by direct assay under anaerobic conditions or with catalase treatment. (C) The inhibition zone diameters of *S*. *aureus* strains were measured. The data are the mean ± SD of three biological independent experiments. Significant differences compared with WT were determined by Dunnett’s test (**, P < 0.01; ***, P < 0.001).

**Fig 2:**
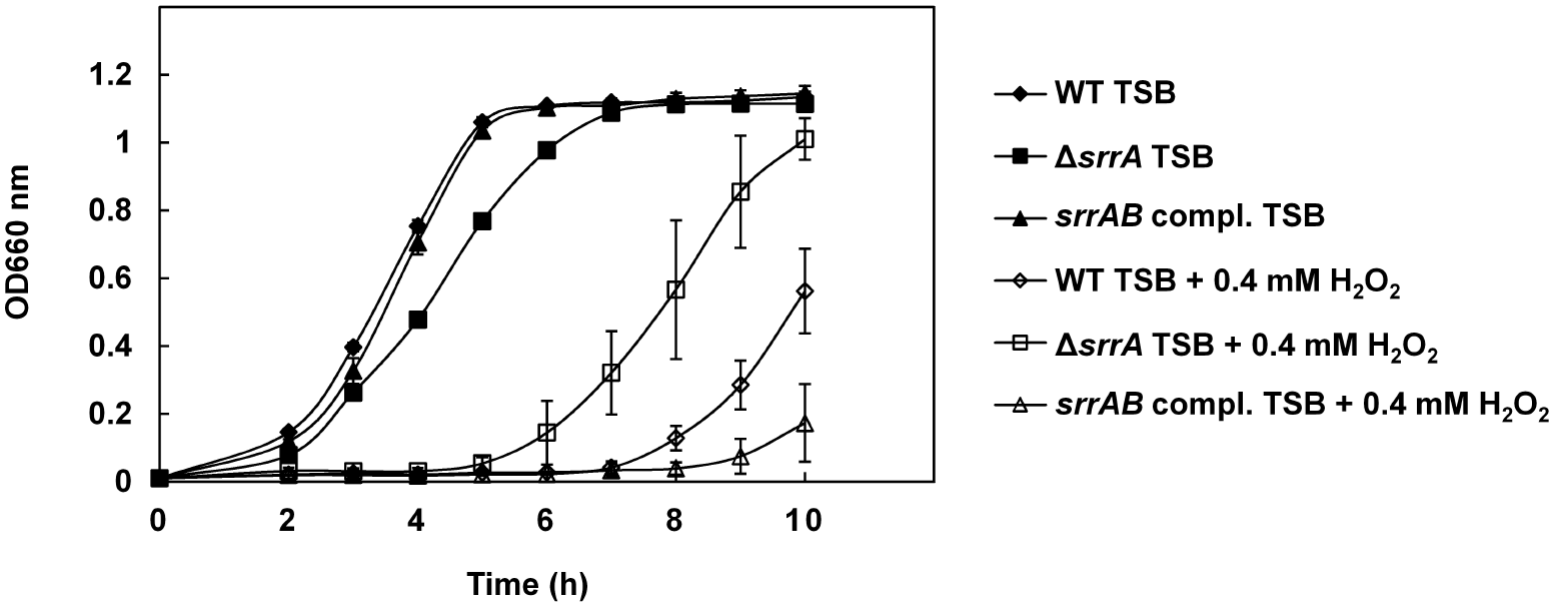
Susceptibility of the *srrA*-inactivated mutant to H_2_O_2_. The bacterial density (OD 660 nm) of *S*. *aureus* MW2 WT, Δ*srrA* and *srrAB* compl. grown in TSB or TSB containing 0.4 mM H_2_O_2_ was measured as described in the Materials and Methods. The data shown represent the means ± SD of three biological independent experiments. Significant differences between the WT and the *srrA*-inactivated mutant grown in TSB containing 0.4 mM H_2_O_2_ were calculated by student’s *t*-test.

A direct assay demonstrated that the *srrA*-inactivated MW2 mutant showed a small inhibition zone surrounding *S*. *sanguinis* compared with the WT and that the small zone of the mutant was restored by complementation with *srrAB* (Fig 1A and 1C). Additionally, we investigated the susceptibility of an *srrA*-inactivated TY34 mutant to *S*. *sanguinis* and found that the mutant had a small inhibition zone compared with the WT (Fig 1C). Under anaerobic conditions, *S*. *aureus* WT showed no inhibition zone, and no inhibition zone was observed after catalase treatment (Fig 1B). In the growth curve experiment, the growth of the *srrA*-inactivated mutant was higher than the WT in the presence of 0.4 mM H_2_O_2_. Statistical significance was observed between WT and the mutant in the presence of 0.4 mM H_2_O_2_ at 10 h incubation. This phenotype in the mutant was restored by complementation with *srrAB* (Fig 2).

### Expression of anti-oxidant factors and *hmp* in the *srrA*-inactivated mutant

**Fig 3:**
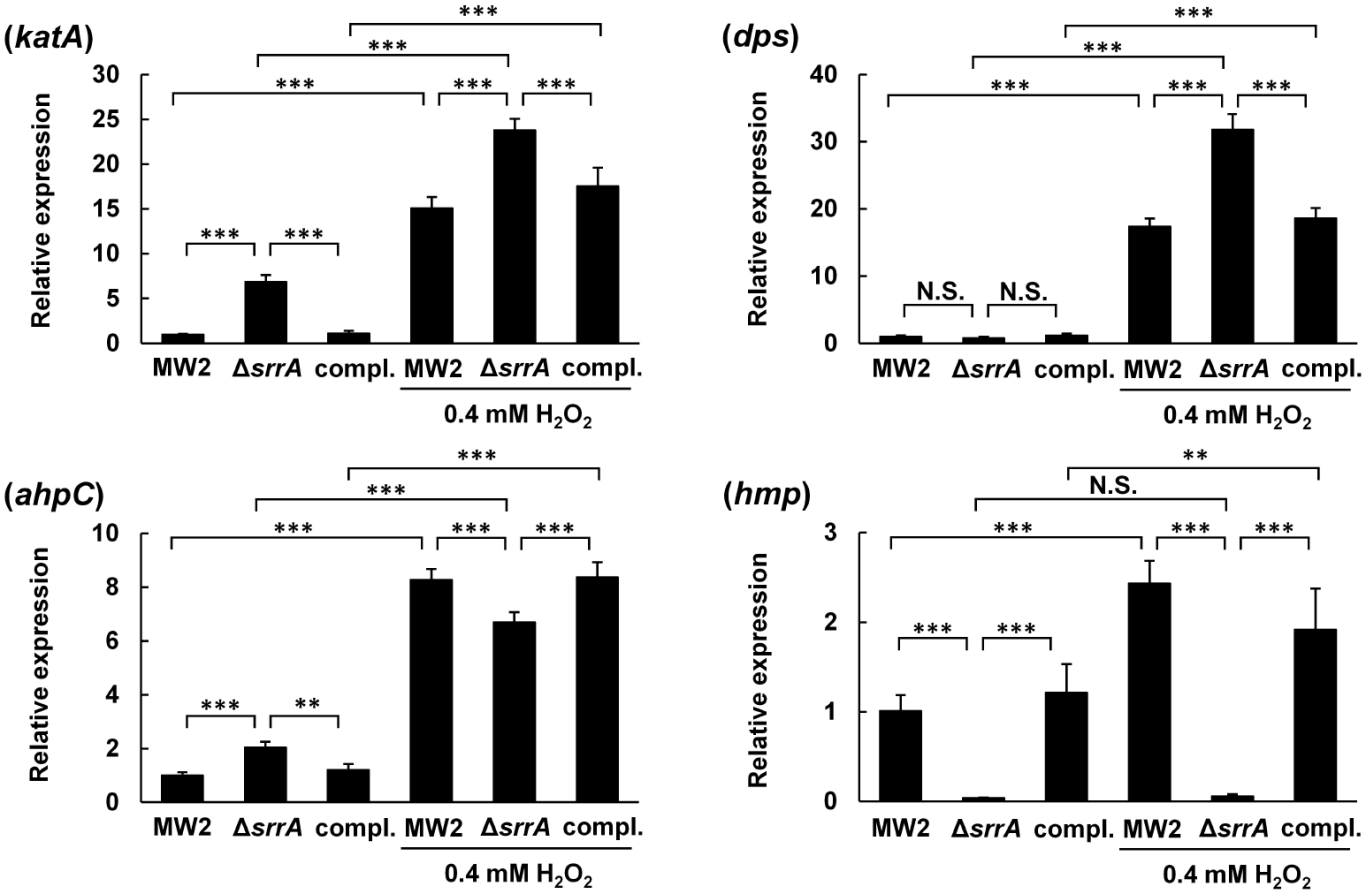
Expression of genes involved in the resistance to oxidative stress and *hmp*. The expression of *katA*, *dps*, *ahpC* and *hmp* in *S*. *aureus* MW2 WT, *srrA*-inactivated mutant and the complemented strain incubated with or without 0.4 mM H_2_O_2_ was determined by quantitative PCR as described in the Materials and Methods section. The data are the mean ± SD of five biological independent experiments. **, *P* < 0.01; ***, *P* < 0.001; N.S., not significant by Tukey's honestly significant difference test.

We used quantitative PCR to investigate the expression of three anti-oxidant factors (*katA*, *dps* and *ahpC*) in the *srrA*-inactivated mutant exposed to 0.4 mM H_2_O_2_ for 10 min. The expression of these three factors in the WT, the *srrA*-inactivated mutant and the complemented strain was increased by H_2_O_2_ treatment. Compared with WT, the expression of *katA* was significantly higher in the mutant in the presence or absence of H_2_O_2_ treatment. The high level was restored in the *srrAB*-complemented strain. The expression of *dps* in the mutant did not increase in the absence of H_2_O_2_, but the expression was significantly higher in the mutant treated with H_2_O_2_. The increased expression in the mutant was restored by complementation. The expression of *ahpC* in the mutant was slightly increased, but the expression was decreased in the mutant compared to the WT when treated with H_2_O_2_ (Fig 3).

Next, we focused on the expression of *hmp* because *hmp* expression is regulated by SrrAB in *S*. *aureus* [34, 35] and is related to oxidative stress in *S*. *enterica* Serovar Typhimurium [44, 45]. The expression of *hmp* in the *srrA*-inactivated mutant was significantly less than in WT treated or untreated with H_2_O_2_. The expression pattern in the complemented strain was similar to that of WT. The expression of *hmp* in the WT was increased 2.4-fold by H_2_O_2_ treatment (Fig 3).

### Susceptibility of H_2_O_2_ and expression of anti-oxidant factors in the *perR*-inactivated mutant

**Fig 4:**
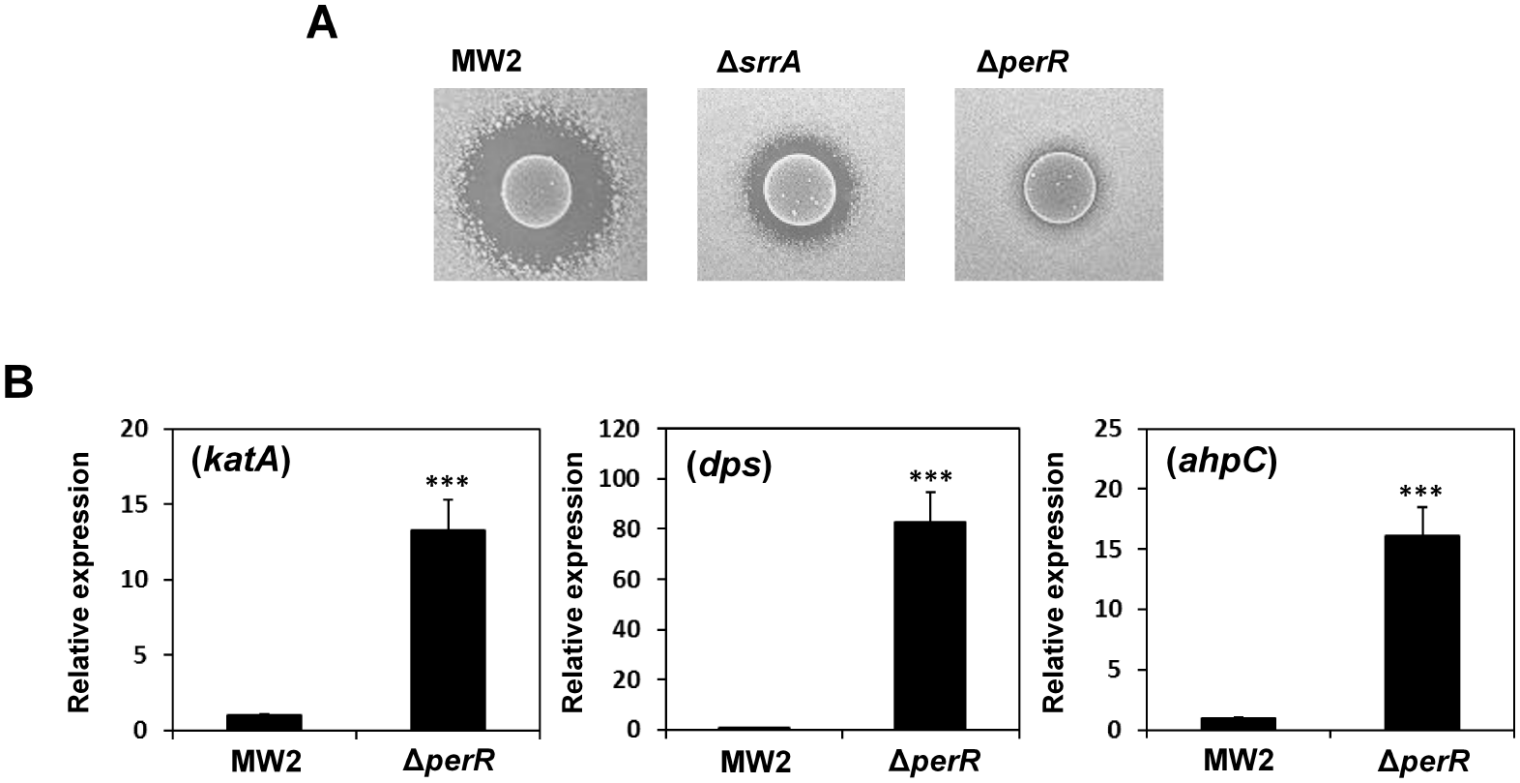
Susceptibility to H_2_O_2_ and expression of *katA*, *dps and ahpC* in the *perR*-inactive mutant. (A) The susceptibilities of *S*. *aureus* MW2, the *srrA*-inactivated mutant and the *perR*-inactivated mutant to the H_2_O_2_ produced by *S*. *sanguinis* were determined by direct assay under aerobic conditions (5% CO_2_). (B) The expression of *katA*, *dps* and *ahpC* in *S*. *aureus* MW2 WT and in the *perR*-inactivated mutant grown in TSB to mid-log phase was determined by quantitative PCR as described in the Materials and Methods section. The data shown represent the mean ± SD of three biological independent experiments. Significant differences compared with WT were determined by Student’s *t*-test (***, P < 0.001).

PerR is related to the regulation of anti-oxidant factors in *S*. *aureus* [46]. We analyzed the susceptibility of the *perR*-inactivated mutant to the H_2_O_2_ produced by *S*. *sanguinis*. As shown in Fig 4A, a *perR*-inactivated mutant strain had significantly lower susceptibility to the H_2_O_2_ produced by *S*. *sanguinis* than the *srrA*-inactivated mutant. The expression of *katA*, *dps* and *ahpC* was significantly increased in the *perR*-inactivated mutant in the absence of H_2_O_2_ (Fig 4B).

### Susceptibility of the *katA*, *dps* or *hmp*-inactivated mutant to H_2_O_2_ produced by *S*. *sanguinis*

**Fig 5:**
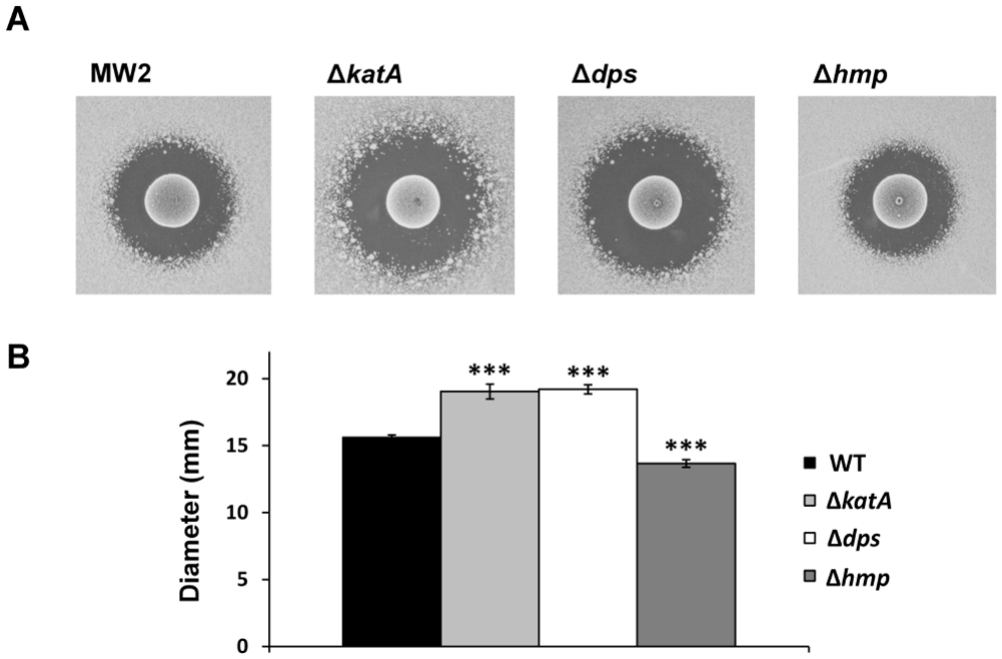
Susceptibility of the *katA*, *dps* or *hmp*-inactivated mutant to H_2_O_2_ produced by *S*. *sanguinis*. (A) The susceptibilities of the *S*. *aureus* MW2 WT, MW2 *katA*, *dps* or *hmp*-inactivated mutants to the H_2_O_2_ produced by *S*. *sanguinis* were analyzed by direct assay, as described in the Materials and Methods section. (B) The diameter of the inhibition zone of *S*. *aureus* strains were measured. The data are the mean ± SD of three biological independent experiments. Significant differences compared with WT were determined by Dunnett’s test (***, P < 0.001).

Because the expression of the two factors (*katA* and *dps*) was increased in the *srrA*-inactivated mutant treated with H_2_O_2_, we constructed a mutant at each locus and performed a direct assay to identify the factor(s) that affected susceptibility to H_2_O_2_. The *katA* and *dps*-inactivated mutants had a large inhibition zone compared with the WT (Fig 5). Additionally, we analyzed the susceptibility of the *hmp*-inactivated mutant to H_2_O_2_, and found that the mutant had a small inhibition zone compared with the WT (Fig 5).

### Co-culture of the *S*. *aureus srrA*-inactivated mutant with *S*. *sanguinis*

**Fig 6:**
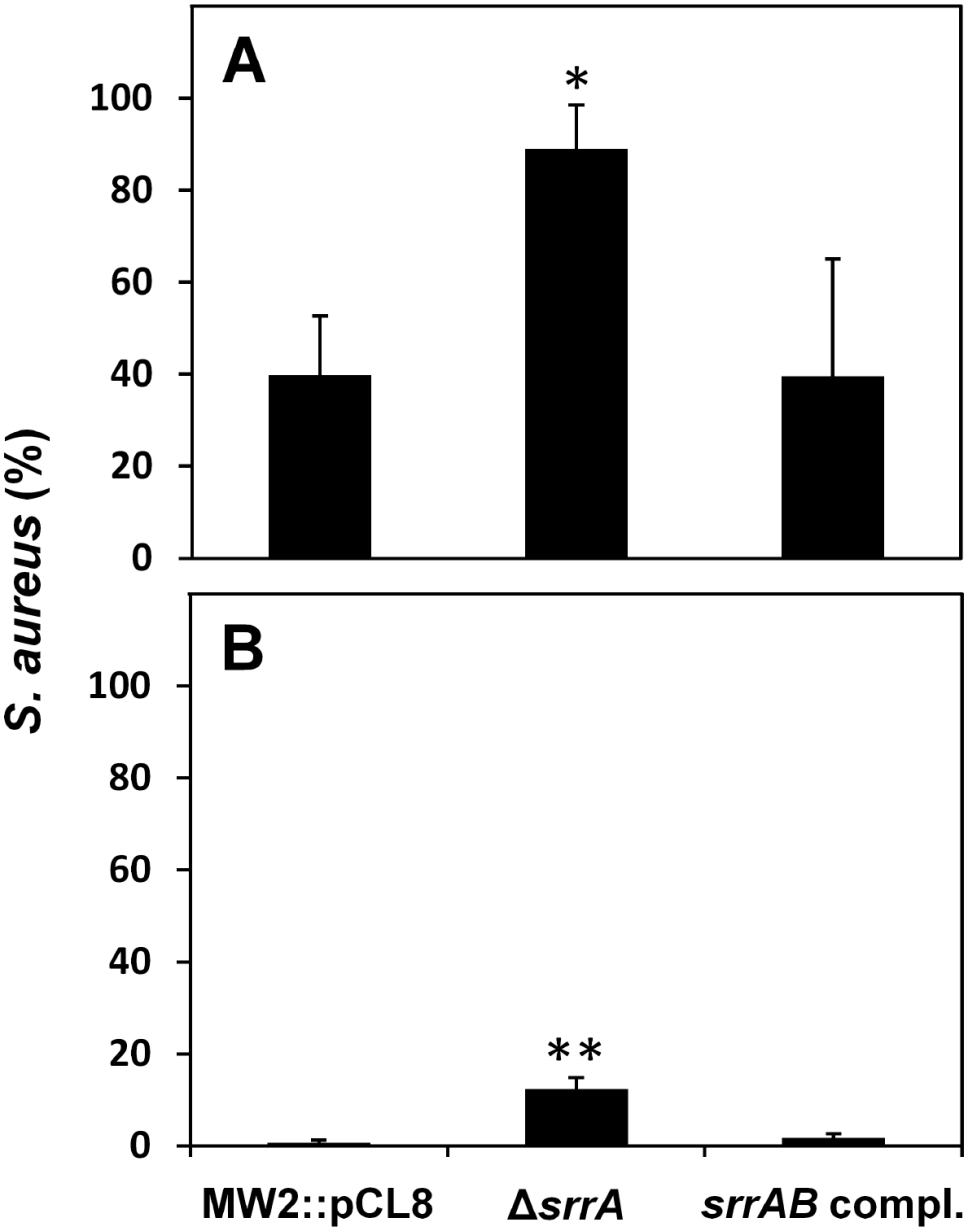
Co-culture of the *srrA*-inactivated mutant with *S*. *sanguinis*. (A) The population percentages of *S*. *aureus* MW2 WT harbouring an empty pCL8 vector (MW2::pCL8), the *srrA*-inactivated mutant and the complemented strain when co-cultured with *S*. *sanguinis* were measured by co-culture assay as described in the Materials and Methods section. (B) The population percentage of MW2 strains co-cultured with pre-cultured (37°C under 5% CO_2_ for 1 h) *S*. *sanguinis*. The data are the mean ± SD of three biological independent experiments. Significant differences compared with WT were determined by Dunnett’s test (*, P < 0.05; **, P < 0.001).

In a preliminary experiment, we demonstrated that the strain MW2 harbouring an empty pCL8 vector (MW2::pCL8) showed an inhibition zone similar to that of strain MW2 with no vector (S1 Fig). Therefore, we used this strain as a WT control for the co-culture assays. Additionally, we analyzed the growth of each *S*. *aureus* strain and *S*. *sanguinis* on TSA plates for 2 h and found that the growth was approximately the same among the *S*. *aureus* strains but that *S*. *sanguinis* grew approximately 2-fold more rapidly compared to the *S*. *aureus* strains (S1 Table). Fig 6A shows the population percentages for the *S*. *aureus* strains co-cultured with *S*. *sanguinis* for 2 h. The mutant population was approximately 2-fold larger than the WT. Fig 6B shows the population percentages of the *S*. *aureus* strains when *S*. *sanguinis* was pre-cultured on a TSA plate for a 1 h. Before the co-culture assay, we demonstrated in a preliminary experiment that the number of *S*. *sanguinis* cells increased 4-fold after 1 h incubation when *S*. *sanguinis* cells alone were spotted on a TSA plate (S1 Table). The mutant population was 18-fold larger than that of the WT.

## Discussion

We demonstrated in this study that an *srrA*-inactivated mutant has a smaller inhibition zone surrounding *S*. *sanguinis* than does the WT by a direct assay and that this inhibition was completely relieved by anaerobic incubation or catalase treatment (Fig 1). In addition, the mutant had a low susceptibility to H_2_O_2_ (Fig 2). Therefore, the small inhibition zone of the *srrA* mutant was caused by the low susceptibility to the H_2_O_2_ produced by *S*. *sanguinis*. Additionally, we demonstrated that the expression of both *katA* and *dps* was increased in the mutant exposed to H_2_O_2_ (Fig 3). Based on these findings, we concluded that the low susceptibility of the *srrA* mutant to H_2_O_2_ was primarily due to the increased expression of *katA* and *dps*.

SrrAB acts as a sensor for low oxygen tension and NO and regulates several factors that facilitate adaptation to these conditions. SrrAB regulates several virulence genes (*tst*, *spa* and *icaA*) under anaerobic or low oxygen conditions [31–33]. The expression of genes involved in anaerobic respiratory pathways (*pflAB*, *adhE* and *nrdDG*), cytochrome assembly and biosynthesis (*qoxABCD*, *cydAB* and *hemABCX*), iron-sulfur cluster repair (*scdA*) and NO detoxification protein (*hmp*) were altered in the *srrAB* mutant under low oxygen or NO stress conditions [34, 35]. Furthermore, phosphatidylinositol-specific phospholipase C (*plc*) was regulated via SrrAB by hypochlorous acid or polymorphonuclear leukocytes [47]. However, the regulation of *katA* and *dps* by SrrAB has not been demonstrated. Recently, Windham *et al*. reported that SrrAB modulates *S*. *aureus* (strain UAMS-1) cell death in high glucose conditions and that an *srrAB* mutant had increased susceptibility to H_2_O_2_. They attributed the increased susceptibility to H_2_O_2_ in the *srrAB* mutant to the production of endogenous reactive oxygen species by the expression of *cidABC* via SrrAB [48]. This report contains results conflicting with our results using *S*. *aureus* strain MW2 and TY34 (Figs 1 and 2). We investigated the expression of *cidA* in the *srrA* mutant of MW2 and found that the expression of *cidA* was significantly repressed by SrrAB (S2 Fig). Therefore, we think that the effect of *cidA* in the *srrA* mutant is not much below the background of MW2 and TY34.

Previously, Horsburgh *et al*. reported that *katA* and *dps* expression in *S*. *aureus* was repressed by PerR, which is a Fur family protein [46]. As shown in Fig 4A, a *perR*-inactivated mutant showed lower susceptibility to H_2_O_2_ than the *srrA*-inactivated mutant. Therefore, we analyzed the relation between SrrAB and PerR. First, we investigated *perR* gene expression in the *srrA*-inactivated mutant and found that *perR* gene expression was unaltered (S3 Fig). Then, we investigated the expression of anti-oxidant factors, and found a higher expression of *katA*, *dps* and *ahpC* in the *perR*-inactivated mutant in the absence of H_2_O_2_ treatment (Fig 4B). Conversely, the increased expression of *dps* was not observed in the *srrA*-inactivated mutant untreated with H_2_O_2_ (Fig 3). These results suggest that the increased expression of *dps* in the *srrA* mutant is not directly related to PerR. PerR is a repressor for several anti-oxidant factors, and this repression was alleviated by H_2_O_2_ [49]. The increased expression of *katA*, *dps* and *ahpC* in the WT and the mutant treated with H_2_O_2_ (Fig 3) indicates that PerR is also involved in the expression of these factors. Compared with the WT, a higher level of *katA* and *dps* transcripts was observed in the *srrA*-inactivated mutant treated with H_2_O_2_ (Fig 3). These results indicate that SrrAB together with PerR is independently involved in *katA* and *dps* regulation. The increased expression of these genes might be an indirect effect of a change in the redox-potential in the *srrA*-inactivated mutant because the mutant showed a decreased expression of the genes responsible for cytochrome assembly and heme biosynthesis in the electron transport chain [35]. However, because the expression pattern of *katA* and *dps* in the mutant was different (Fig 3), further studies will be required to clarify the link between SrrAB and *katA* or *dps*.

In addition, we demonstrated for the first time that Hmp was associated with H_2_O_2_ susceptibility in *S*. *aureus*. A relationship between Hmp and susceptibility to oxidative stress has been reported in *E*. *coli* and *S*. *typhimurium* [50, 44]. In the presence of NO, Hmp converts NO to nitrate (NO_3_^-^) by the reaction NO + O_2_ + e^-^ → NO_3_^-^ utilizing an electron from the reduction of flavin adenine dinucleotide (FAD) [51]. In the absence of NO, Hmp has the potential to generate superoxide anion radicals (O_2_^-^) by the reaction O_2_ + e^-^ → O_2_^-^ utilizing an electron from the reduction of FAD [52]. In addition, Hmp is associated with the production of ·OH from H_2_O_2_ via the Fenton chemistry in the absence of NO [44]. Based on these reports, it is thought that *hmp* inactivation in *S*. *aureus* suppresses the generation of intracellular oxidative stress, and the mutant showed lower susceptibility to H_2_O_2_ than the WT. NsrR, which is a Rrf2 family transcription repressor, was demonstrated to repress the generation of oxidative stress in the absence of NO by repressing the expression of *hmp* in several bacterial species, including *E*. *coli*, *S*. *typhimurium* and *B*. *subtilis* [53]. The inactivation of *nsrR* results in high susceptibility to H_2_O_2_ in *S*. *typhimurium* [45]. However, we could not find the gene *nsrR* or an *nsrR* homologue in the *S*. *aureus* genome database. TCS, SrrAB and/or ResDE have been reported to regulate Hmp in the presence of NO in *S*. *aureus* and *Bacillus subtilis* [34, 35, 54]. In *B*. *subtilis*, ResDE regulates Hmp expression in an NsrR-dependent manner [55], whereas in *S*. *aureus*, Hmp was dependent on SrrAB regulation. We suggest that the expression of *hmp* is regulated by SrrAB and affects the susceptibility to H_2_O_2_.

In a co-culture assay, the percentage of the *srrA*-inactivated mutant was high in a mixed culture with *S*. *sanguinis*. Because several oral streptococci, such as *S*. *sanguinis*, *S*. *parasanguinis*, *S*. *gordonii* and *S*. *oralis*, can produce H_2_O_2_ [56–58], *S*. *aureus* requires H_2_O_2_ resistance to survive in the oral cavity. In the oral cavity, *S*. *aureus* can colonize under anaerobic (dental plaque and gingival sulcus) and aerobic conditions (oral mucosa) [8–10]. Therefore, *S*. *aureus* can modulate its susceptibility to H_2_O_2_ by SrrAB activity and coexist with H_2_O_2-_producing oral streptococci, including *S*. *sanguinis*. Further studies will be required to analyze the functions of SrrAB involved in the co-existence with H_2_O_2_-producing bacteria in vivo, particularly in the oral cavity.

## Supporting Information

S1 Fig Susceptibility of MW2 harbouring the empty pCL8 to H_2_O_2_ produced by *S*. *sanguinis*. The susceptibilities of *S*. *aureus* MW2 and MW2 harbouring an empty pCL8 vector (MW2::pCL8) to H_2_O_2_ produced by *S*. *sanguinis* were determined by direct assay under aerobic conditions (5% CO_2_).
(TIF)

S2 Fig Expression of *cidA* in *srrA*-inactivated mutant. The expression of *cidA* in mid-log phase (cell density at 660 nm = 0.8) cells of *S*. *aureus* MW2 WT, *srrA*-inactivated mutant and the complemented strain grown in TSB was determined by quantitative PCR as described in the Materials and Methods section. The data are the mean ± SD of five biological independent experiments. Significant differences compared with WT were determined by Dunnett’s test (***, P < 0.001; N.S., not significant).
(TIF)

S3 Fig Expression of *perR* in *srrA*-inactivated mutant. The expression of *perR* in mid-log phase (cell density at 660 nm = 0.8) cells of *S*. *aureus* MW2 WT, *srrA*-inactivated mutant and the complemented strain grown in TSB was determined by quantitative PCR as described in the Materials and Methods section. The data are the mean ± SD of five biological independent experiments. Significant differences compared with WT were determined by Dunnett’s test (N.S., not significant).
(TIF)

S1 Table Bacterial growth of *S*. *sanguinis* and *S*. *aureus* strains on TSA plates. (DOCX)
