# Supplementary figures and images for: Staphylococcus aureus SrrAB Affects Susceptibility to Hydrogen Peroxide and Co-Existence with Streptococcus sanguinis
Source: PLoS One. 2016 Jul 21;11(7):e0159768. doi: 10.1371/journal.pone.0159768 (PMC4956065; doi:10.1371/journal.pone.0159768)

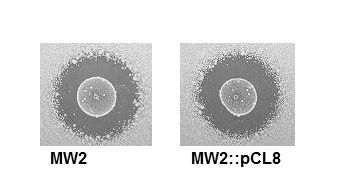

Supplement: S1 Fig — The susceptibilities of S. aureus MW2 and MW2 harbouring an empty pCL8 vector (MW2::pCL8) to H2O2 produced by S. sanguinis were determined by direct assay under aerobic conditions (5% CO2). (TIF) [file pone.0159768.s001.tif]

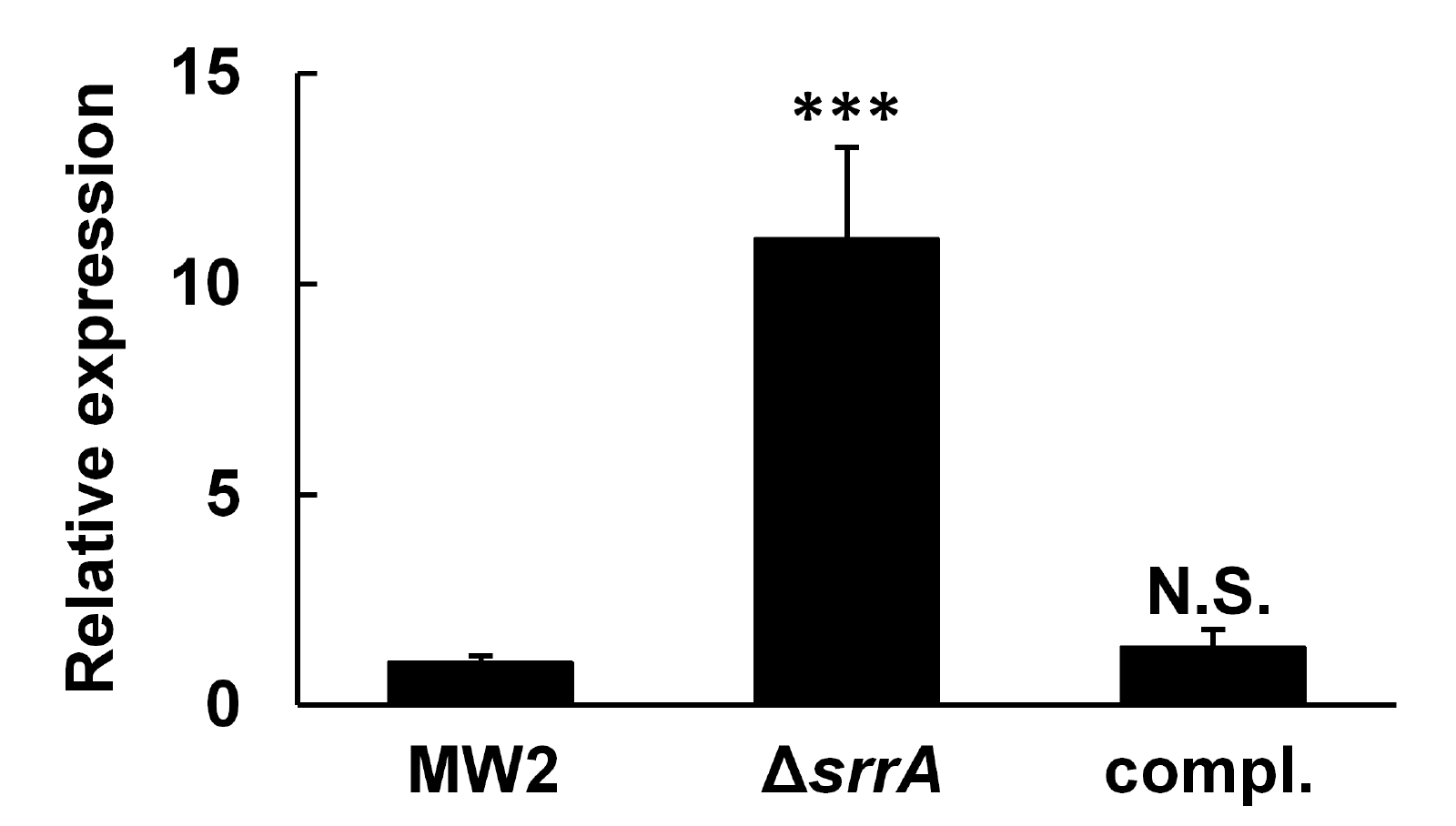

Supplement: S2 Fig — The expression of cidA in mid-log phase (cell density at 660 nm = 0.8) cells of S. aureus MW2 WT, srrA-inactivated mutant and the complemented strain grown in TSB was determined by quantitative PCR as described in the Materials and Methods section. The data are the mean ± SD of five biological independent experiments. Significant differences compared with WT were determined by Dunnett’s test (***, P < 0.001; N.S., not significant). (TIF) [file pone.0159768.s002.tif]

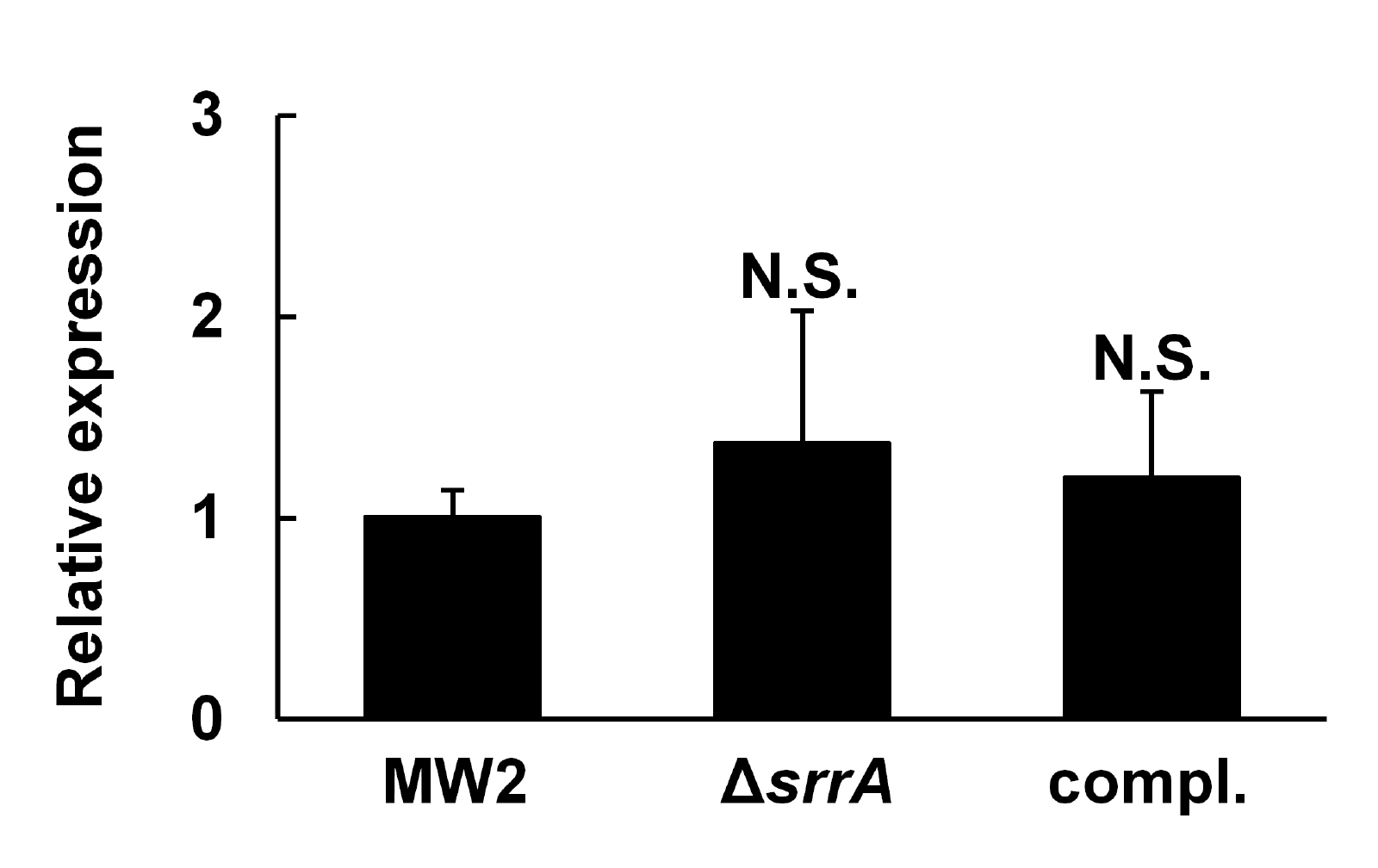

Supplement: S3 Fig — The expression of perR in mid-log phase (cell density at 660 nm = 0.8) cells of S. aureus MW2 WT, srrA-inactivated mutant and the complemented strain grown in TSB was determined by quantitative PCR as described in the Materials and Methods section. The data are the mean ± SD of five biological independent experiments. Significant differences compared with WT were determined by Dunnett’s test (N.S., not significant). (TIF) [file pone.0159768.s003.tif]
