# Supplementary material for: Staphylococcus aureus SrrAB Affects Susceptibility to Hydrogen Peroxide and Co-Existence with Streptococcus sanguinis
Source: PLoS One. 2016 Jul 21;11(7):e0159768. doi: 10.1371/journal.pone.0159768 (PMC4956065; doi:10.1371/journal.pone.0159768)
Supplement: S1 Table — (DOCX) [file pone.0159768.s004.docx]

**Table S1** Bacterial growth of *S. sanguinis* and *S. aureus* strains on TSA plates

| Strains |  | CFU (×10^4^) *^a^* | | |
| --- | --- | --- | --- | --- |
|  |  | 0 h | 1 h | 2 h |
| *S. sanguinis* GTC217 |  | 118.7 ± 12.5 | 436.7 ± 15.3 | 1207 ± 168.6 |
| *S. aureus* MW2::pCL8 |  | 116.3 ± 6.5 | N.D. *^b^* | 710 ± 90.4 |
| *S. aureus* MW2 Δ*srrAB* |  | 104.3 ± 8.1 | N.D. | 615 ± 79.4 |
| *S. aureus* MW2 *srrAB* compl. |  | 92.3 ± 12.5 | N.D. | 640 ± 87.9 |

*^a^* Total amount of 10^6^ cell of *S. sanguinis* and *S. aureus* (MW2 WT harbouring an empty pCL8 vector, *srrA*-inactivated mutant and the complemented strain) was dropped onto a TSA plate. The plate was incubated for 1 or 2 h at 37 °C under 5 % CO_2_. The agar in the spotted area was excised and incorporated into 500 µl of PBS. Then, the agar was vigorously mixed to detach the bacterial cells from the agar. Appropriate dilutions were plated on TSA plates. After an overnight incubation at 37 °C under 5 % CO_2_, CFUs were determined. The data are the mean ± SD of three biological independent experiments.

*^b^* No data
